# Clinical characteristics of heart failure associated with functional dependence at admission in hospitalized elderly

**DOI:** 10.1590/1518-8345.2869-3137

**Published:** 2019-04-29

**Authors:** Sara de Oliveira Xavier, Renata Eloah de Lucena Ferretti-Rebustini

**Affiliations:** 1 Universidade de São Paulo Escola de Enfermagem São Paulo, SP, Brasil .

**Keywords:** Heart Failure, Dependence, Elderly, Activities of Daily Living, Hospitalization, Nursing, Insuficiência Cardíaca, Dependência, Idoso, Atividades Básicas de Vida Diária, Hospitalização, Enfermagem, Insuficiencia Cardiaca, Dependiente, Anciano, Actividades de la Vida Diaria, Hospitalización, Enfermería

## Abstract

**Objective:**

to identify which clinical features of heart failure are associated with a greater chance of functional dependence for the basic activities of daily living in hospitalized elderly.

**Method:**

cross-sectional study conducted with elderly hospitalized patients. The clinical characteristics of heart failure were assessed by self-report, medical records and scales. Dependency was assessed by the Katz Index. The Fisher’s Exact Test was used to analyze associations between the nominal variables, and logistic regression to identify factors associated with dependence.

**Results:**

the sample consisted of 191 cases. The prevalence of functional dependence was 70.2%. Most of the elderly were partially dependent (66.6%). Clinical characteristics associated with dependence at admission were dyspnea (Odds Ratio 8.5, Confidence Interval 95% 2.668-27.664, p <0.001), lower limb edema (Odds Ratio 5.7, 95% Confidence Interval 2.148-15.571, p <0.001); cough (Odds Ratio 9.0, 95% confidence interval 1.053-76.938, p <0.045); precordial pain (Odds Ratio 4.5, 95% confidence interval 1.125-18.023, p <0.033), and pulmonary crackling (Odds Ratio 4.9, 95% Confidence Interval 1.704-14.094, p <0.003).

**Conclusion:**

functional dependence in admitted elderly patients with heart failure is more associated with congestive signs and symptoms.

## Abstract (pt)

**Objetivo:**

identificar quais características clínicas da insuficiência cardíaca estão associadas à maior chance de dependência funcional admissional para as atividades básicas de vida diária em idosos hospitalizados.

**Método:**

estudo transversal realizado com idosos hospitalizados. As características clínicas da insuficiência cardíaca foram avaliadas por meio do autorrelato, prontuário e aplicação de escalas. A dependência foi avaliada pelo Índice de Katz. Realizou-se o Teste Exato de Fisher para análise das associações entre as variáveis nominais e regressão logística para identificação de fatores associados à dependência.

**Resultados:**

a amostra foi composta por 191 casos. A prevalência de dependência funcional admissional foi 70,2%. Grande parte dos idosos era parcialmente dependente (66,6%). As características clínicas associadas à dependência admissional foram: dispneia (Odds Ratio 8,5; Intervalo de confiança 95% 2,668-27,664; p<0,001), edema de membros inferiores (Odds Ratio 5,7; Intervalo de confiança 95% 2,148-15,571; p<0,001); tosse (Odds Ratio 9,0; Intervalo de confiança 95% 1,053-76,938; p<0,045); precordialgia (Odds Ratio 4,5; Intervalo de confiança 95% 1,125-18,023; p<0,033) e crepitação pulmonar (Odds Ratio 4,9; Intervalo de confiança 95% 1,704-14,094; p<0,003).

**Conclusão:**

observou-se que a dependência funcional admissional em idosos com insuficiência cardíaca está mais associada com sinais e sintomas congestivos.

## Abstract (es)

**Objetivo:**

identificar cuáles son las características clínicas de la insuficiencia cardíaca que están asociadas a la mayor chance de dependencia funcional en el proceso de admisión para las actividades básicas de vida diaria en adultos mayores hospitalizados.

**Método:**

estudio transversal realizado con adultos mayores hospitalizados. Las características clínicas de la insuficiencia cardíaca fueron evaluadas por medio del auto relato, prontuario y aplicación de escalas. La dependencia fue evaluada por el Índice de Katz. Se realizó el Test Exacto de Fisher para análisis de las asociaciones entre las variables nominales y regresión logística para la identificación de factores asociados a la dependencia.

**Resultados:**

la muestra fue compuesta por 191 casos. La prevalencia de dependencia funcional en el proceso de admisión fue 70,2%. Gran parte de los adultos mayores eran parcialmente dependientes (66,6%). Las características clínicas asociadas a la dependencia fueron: disnea (Odds Ratio 8,5; Intervalo de confianza 95% 2,668-27,664; p<0,001), edema de miembros inferiores (Odds Ratio 5,7; Intervalo de confianza 95% 2,148-15,571; p<0,001); tos (Odds Ratio 9,0; Intervalo de confianza 95% 1,053-76,938; p<0,045); precordialgia (Odds Ratio 4,5; Intervalo de confianza 95% 1,125-18,023; p<0,033) y crepitación pulmonar (Odds Ratio 4,9; Intervalo de confianza 95% 1,704-14,094; p<0,003).

**Conclusión:**

se observó que la dependencia funcional de los adultos mayores con insuficiencia cardíaca está más asociada con señales y síntomas congestivos.

## Introduction

With the aging of the world population, there has been a progressive increase of Cardiovascular Diseases (CVD), including Heart Failure (HF). This is a common condition in the elderly and an increase in its incidence and prevalence is estimated to occur in the coming years, becoming a serious public health problem ^(^ ^1^ ^-^ ^2^ ^)^ . Projections show that the prevalence of HF will increase 46% by the year 2030, resulting in more than 8 million cases ^(^ ^3^ ^)^ . The most comprehensive picture of the situation of hospitalizations due to HF in Brazil can be obtained through the analyzes of the records of the Department of Informatics of the Unified Health System (DATASUS), in which 23,833 deaths were attributed to HF in the elderly only in the year 2015 ^(^ ^4^ ^)^ .

HF is a clinical syndrome in which a structural or functional change in the heart leads to the inability to eject or accommodate blood within physiological pressure values, causing functional limitation and the need for therapeutic intervention ^(^ ^5^ ^)^ .

In hospitalized elderly, the clinical picture is complex and influenced by the presence of an arsenal of signs and symptoms ^(^ ^6^ ^)^ , which are described as clinical characteristics of HF and represent a high risk for dependence, hospital readmissions, morbidity and mortality ^(^ ^7^ ^)^ .

The clinical characteristics of HF are represented by the symptoms of fatigue, dyspnea, lower limb edema, cough, precordial pain, dizziness, palpitation, orthopnea and paroxysmal nocturnal dyspnea; and by signs of crackling at lung auscultation, jugular stasis, signs of hepatomegaly, ascites, and left ventricular ejection fraction (LVEF). In the elderly, dyspnea and fatigue are prominent, which may contribute to exercise intolerance and culminate in dependence on the Basic Activities of Daily Living (BADL) ^(^ ^8^ ^-^ ^9^ ^)^ . The situation becomes more evident during hospitalization in which even the independent elderly may need help in BADL, which makes the individual more susceptible to dependence and loss of autonomy ^(^ ^10^ ^-^ ^12^ ^)^ .

Hospitalization due to HF, therefore, is considered a marker of clinical instability ^(^ ^13^ ^)^ and is associated with an increase in patient dependence. It is estimated that 25 to 35% of the hospitalized elderly will present some functional impairment after discharge ^(^ ^14^ ^)^ and there is a high risk of potentiating an already existing functional decline ^(^ ^15^ ^)^ . The time of hospitalization for clinical compensation is an important aspect, since it implies costs. The more clinically severe patients, with more comorbidities and, consequently, those with the highest number of associated clinical characteristics, need more time for compensation and will cost more for the health system ^(^ ^16^ ^)^ .

Thus, the identification of individuals at greater risk of functional decline should be a routine action in nursing care practice, since it may contribute to minimize the adverse consequences of hospitalization. Therefore, individualized nursing actions will meet the care demands according to the functional performance of the elderly ^(^ ^17^ ^)^ .

Although age progression may naturally influence functionality, there are not many studies in the literature exploring the association between Functional Dependence (FD) and HF. A previous study showed that HF is associated with higher FD at hospital admission ^(^ ^17^ ^),^ however, it did not explore which component of the HF is associated with the dependence state. No studies were found on whether clinical features of HF are associated with FD in elderly hospitalized patients.

Therefore, the present study aimed to identify the clinical characteristics of HF associated with FD for BADL at admission in hospitalized elderly.

## Methods

This is an epidemiological, observational and cross-sectional study. The data were collected in nursing wards with beds destined for hospitalization of cardiac patients of a reference hospital in cardiology in the city of São Paulo, Brazil. The study was approved by the Local Ethics Committee (CAAE 62435716.7.0000.5392), after consent of the hospital institution.

The sample for convenience was estimated in 144 cases (by means of a sample calculation, with a statistical power of 95% and a confidence level of 95%). To predict losses, 33% of additional cases were collected. All cases were included in the study after obtaining the signatures of the Informed Consent Form.

The inclusion criteria were being aged ≥ 60 years on admission, having a medical diagnosis of HF and being available for evaluation in the first 24 hours of hospitalization. All the elderly admitted to the hospital from July 2017 to February 2018 and who met the said criteria were included in the study. Individuals with total dependence for BADL prior to hospitalization were excluded.

The data were collected in the first 24 hours of hospitalization of the elderly in the unit through a clinical interview with application of evaluation scales and through consultation in medical records. Each interview lasted an average of 20 minutes. In the interview with the elderly, the demographic and clinical information regarding the presence or absence of HF characteristics was collected. The ‘fatigue’ symptom was assessed by applying the Dutch Exertion Fatigue Scale (DEFS) ^(^ ^18^ ^)^ , given its subjectivity. Data on LVEF and Functional Class of the New York Heart Association (FC-NYHA) were extracted from the medical records.

The functional evaluation for BADL was performed through the application of the Katz Index ^(^ ^19^ ^)^ . The instrument was applied twice during the interview: the first application was done retrospectively, with reference to the week prior to hospitalization (prior FD) and the second was done with reference to the time of hospital admission (the first 24 hours - FD at admission).

Data were analyzed using SPSS software, v.22. Descriptive analyzes were performed presenting absolute and relative frequencies; means, standard deviation, medians, and variation (minimum and maximum). The Fisher’s Exact Test was performed to analyze the associations between the nominal variables.

For the identification of factors associated with FD, logistic regression was performed. The model included the dependent variable (FD at admission) and the independent variables, which were the clinical characteristics of HF (presence of fatigue, dyspnea, lower limb edema, cough, precordial pain, dizziness, palpitation, paroxysmal nocturnal dyspnea, orthopnea, crackling at lung auscultation, signs of hepatomegaly, ascites, jugular stasis and reduced LVEF). The Odds Ratio (OR) value was presented with its 95% confidence interval and significant p-value (≤0.05).

## Results

The sample consisted of 191 elderly, mostly males (n = 106, 55.5%). Mean age was 75.6 years (SD = 9.1) and women were on average about two years older than men, but this difference was not statistically significant (p <0.082). The majority of the sample consisted of white individuals (77.5%), retired (61.8%) and married (53.9%). Among women, the mean number of years of study was 4.2 (SD = 2.4) and among men, 5.4 (SD = 2.6). Men had 1.2 year of schooling more than women (p <0.001).

For most participants (n = 98; 51.3%), HF had been diagnosed for more than 21 years. Regarding the functional clinical characterization of HF, there was a predominance of Functional Class III (53.4%). Decompensated HF was a frequent cause of hospitalization, accounting for 65 (34%) admissions.

In relation to previous FD, 70 (36.6%) elderly were partially dependent, while 121 (63.4%) were independent for BADL in the week prior to hospital admission. At hospital admission, most of the elderly (n = 134; 70.2%) presented functional dependence at admission and there was no evidence of an association between functional loss and the previous functional status (p <0.212). On the day of admission, 7 (3.7%) elderly were totally dependent, 127 (66.6%) were partially dependent and 57 (29.7%) were independent for the BADL. There was an association between hospitalization due to decompensated HF and FD (p <0.001).

Concerning clinical characteristics, dyspnea (n = 164, 85.9%), paroxysmal nocturnal dyspnea (n = 123; 64.4%), palpitation (n = 88, 46.1%) and fatigue (n = 106; 44.5%) were the most prevalent symptoms in the sample. Other signs and symptoms presented by the elderly were lower limb edema (n = 77, 40.3%), orthopnea (n = 68, 35.6%), cough (n = 29, 15.2%), precordial pain (n = 21, 11.0%), dizziness (n = 13, 6.8%), crackling at lung auscultation (n = 76, 39.8%), jugular stasis (n = 30, 15.7%), hepatomegaly (n = 13, 6.8%) and ascites (n = 5, 2.6%).

In relation to LVEF, the highest frequencies were observed between reduced LVEF (n = 78, 40.8%) and preserved LVEF (n = 77, 40.3%), respectively, while 18.9% had borderline LVEF (n = 36). Table 1 shows the clinical characteristics of HF as a function associated with the functional profile, showing a higher frequency of FD among individuals with congestive signs and symptoms.

**Table 1:** – Association between clinical characteristics of the Heart Failure and Functional Profile at admission in the elderly (n = 191). São Paulo, SP, Brazil, 2018

**Clinical features**		**Functional profile at admission [n (%)]**				

		**Dependent**	**Partially dependent**	**Independent**	**Total [n (%)]**	*p-* value*
Fatigue	Yes	3 (2.8)	73 (68.8)	30 (28.4)	106 (55.4)	0.109
	No	4 (2.1)	54 (28.3)	27 (14.1)	85 (44.6)	
Dyspnea	Yes	6 (3.6)	117 (71.4)	41 (25.0)	164 (85.8)	0.001
	No	1 (3.7)	10 (37.0)	16 (59.3)	27 (14.2)	
Edema of LL ^†^	Yes	5 (6.5)	62 (80.5)	10 (13.0)	77 (40.3)	0.001
	No	2 (1.7)	65 (57.0)	47 (41.3)	114 (59.7)	
Cough	Yes	1 (3.4)	27 (93.2)	1 (3.4)	29 (15.1)	0.002
	No	6 (3.7)	100 (61.8)	56 (34.5)	162 (84.9)	
Precordial pain	Yes	0 (0)	17 (80.9)	4 (19.1)	21 (11.0)	0.144
	No	7 (4.1)	110 (64.7)	53 (31.2)	170(89.0)	
Dizziness	Yes	1 (7.7)	8 (61.5)	4 (30.8)	13 (6.8)	0.216
	No	6 (3.4)	119 (66.8)	53 (29.8)	178 (93.2)	
Palpitation	Yes	0 (0)	10 (55.5)	8 (44.5)	18 (9.4)	0.056
	No	7 (4.0)	117 (67.6)	49 (28.4)	173 (90.6)	
Orthopnea	Yes	6 (4.8)	74 (60.2)	43 (35.0)	123 (64.3)	0.044
	No	1 (1.5)	53 (78.0)	14 (20.5)	68(35.7)	
PND ^‡^	Yes	2 (2.3)	59 (67.0)	27 (30.7)	88 (46.0)	0.096
	No	5 (4.9)	68 (66.0)	30 (29.1)	103(54.0)	
Crackling	Yes	4 (5.3)	64 (84.2)	8 (10.5)	76 (39.8)	0.001
	No	3 (2.6)	63 (54.7)	49 (42.7)	115(60.2)	
Jugular stasis	Yes	1 (3.3)	26 (86.7)	3 (10.0)	30 (15.7)	0.012
	No	6 (3.7)	101 (62.7)	54 (33.6)	161 (84.3)	
Hepatomegaly	Yes	0 (0)	11 (84.6)	2 (15.4)	13 (6.8)	0.167
	No	7 (4.0)	116 (65.1)	55 (30.9)	178 (93.2)	
Ascites	Yes	0 (0)	4 (80.0)	1 (20.0)	5 (2.6)	0.338
	No	7 (3.8)	123 (66.1)	56 (30.1)	186 (97.4)	
RLVEF ^§^	Yes	0 (0.0)	59 (74.7)	20 (25.3)	79 (41.4)	0.011
	No	8 (7.1)	67 (59.8)	37 (33.1)	112 (58.6)	

*p-value = Fisher’s exact test; †LL = Lower limbs; ‡PND = paroxysmal nocturnal dyspnea; §RLVEF = Reduced left ventricular ejection fraction

Table 2 presents the factors associated with the greater chance of FD at admission in elderly with HF.

**Table 2:** – Factors associated with functional dependence in elderly patients with heart failure (n = 191). São Paulo, SP, Brazil, 2018

Clinical features	OR*	CI ^†^ (95%)	*p* -value ^‡^
Fatigue	1.076	0.997-1.160	0.059
Dyspnea	8.591	2.668-27.664	0.001
Lower limb edema	5.784	2.148-15.571	0.001
Cough	9.000	1.053-76.938	0.045
Precordial pain	4.503	1.125-18.023	0.033
Dizziness	1.958	0.375-10.219	0.425
Palpitation	0.348	0.090-1.348	0.127
Orthopnea	0.265	0.104-0.677	0.005
Paroxysmal nocturnal dyspnea	1.277	0.568-2.872	0.554
Pulmonary crackling	4.900	1.704-14.094	0.003
Jugular stasis	1.920	0.337-10.929	0.462
Hepatomegaly	0.964	0.118-7.910	0.973
Ascites	0.085	0.006-1.149	0.064
Reduced ejection fraction	0.749	0.490-2.693	1.149

*OR = odds ratio; †CI = confidence interval; ‡ *p* -value = Fisher’s exact test

Thus, the hemodynamic profile with congestive pattern is associated with a greater chance of FD.

## Discussion

The results of the present study showed a high prevalence of FD at admission. The symptoms of dyspnea, PND, palpitation and fatigue were the most frequent, but the clinical characteristics associated to the greater chance of FD at admission were dyspnea, edema of LL, cough, precordial pain and pulmonary crackling.

In a comprehensive way, congestive and respiratory signs and symptoms are directly associated with FD and unfavorable outcomes ^(^ ^20^ ^-^ ^21^ ^)^ . Studies indicate that patients with HF and those with volume overload represent the largest contingent in their decompensated form ^(^ ^22^ ^)^ .

Respiratory symptoms are understood to be threatening ^(^ ^23^ ^)^ and dyspnea, in particular, is the predominant symptom in this population.

The dyspnea symptom is worth noting in the sample, as it not only presented high prevalence (85.9%), association with the outcome of dependence, male sex and age group of 80 years or older, but it was also strongly associated with higher FD in as it was responsible for increasing the chance of FD by up to 8.5 times. It is evident that such a symptom may have a strong impact on hospitalization and its relationship with FD, with repercussions on BADL during hospitalization and after discharge. One of the predisposing factors for dyspnea in HF patients in other investigations is the deterioration in the skeletal muscles in the lower and upper limbs ^(^ ^24^ ^)^ and in the respiratory muscles, triggering limiting symptoms and functional loss ^(^ ^25^ ^-^ ^26^ ^)^ .

Potentially, in this research, limb edema was considered an associated factor, increasing the chance of FD by up to 5.7 times; however, previous studies have not clearly described this association, making it difficult to compare the results. Therefore, edema in the lower limbs may cause mobility restriction, hindering gait due to increased interstitial fluid volume deriving from hemodynamic failure.

Another clinical characteristic associated with a greater chance of FD was precordial pain, which may increase the risk of functional losses by 4.5 times. In this regard, the elderly may choose to spend less energy in order to prevent episodes of stress-related precordial pain, culminating in less active individuals. This prediction should be understood as an impact factor and demands more evidence based on prospective studies.

Cough was considered a factor associated with FD (OR = 9.0) in this study. In addition, it had an association with being a male and between 70 and 79 years of age. Also, no studies were found that analyzed this association, despite the strong evidence between congestive symptoms and functional loss, which may be a consequence of pulmonary overload due to left ventricular failure ^(^ ^27^ ^)^ . In the literature, there are only reports of prevalence of cough as a symptom in the population with HF ^(^ ^28^ ^)^ .

Although much of the signs and symptoms have not been associated with a greater chance of FD, these data cannot be fully conclusive or discarded, and new studies should be encouraged to be analyzed with caution. The clinical characteristics of HF were assessed through self-report of patients, except for fatigue and reduced LVEF. Thus, the elderly may have underestimated some symptom. Some recurrent symptoms in the elderly with HF, such as fatigue, dyspnea and orthopnea, are often erroneously interpreted as deriving from the aging process during hospital admission or underestimated.

So, the elderly can undergo an adaptive process about the signs and symptoms of HF and can, therefore, face them with tolerance in day-to-day activities. In addition, the signs and symptoms of HF in the elderly may manifest atypically, according to the peculiar characteristics of aging.

Fatigue, in this study, was not considered a predictor of FD, contrary to other studies. It has been demonstrated that fatigue has a high incidence in elderly with HF, being associated with DF and deserving to be studied in other investigations ^(^ ^29^ ^)^ . In spite of the wide incidence of fatigue, its evaluation is carried out in a diversified way due to the range of measurement instruments, which requires attention to integrate information about this clinical characteristic. Authors suggest the establishment of cutoff points for the evaluation instruments of fatigue in the elderly ^(^ ^30^ ^)^ .

According to the Cardiac Rehabilitation Directive, published in 2005, the onset of fatigue and dyspnea during exercise limits the performance of BADL, reducing quality of life. In addition, elderly patients present with neurohumoral exacerbation and increased ventilatory response during exercise, which limits functional ability ^(^ ^31^ ^)^ . Symptoms of PND and orthopnea are frequently reported by patients with HF, which impairs sleep quality in this population and may result in energy changes that favor dependence.

Over the last few decades, an exponential number of studies have demonstrated the pathophysiological mechanisms of HF and new pharmacological therapies have been continually discovered. However, despite considerable progress in the therapeutic management of elderly patients with HF, mortality and morbidity remain a major concern, and hospital admissions compromise performance in BADL ^(^ ^32^ ^)^ . Thus, recognizing which factors are associated with a greater chance of FD in the elderly with HF may be the differential for improving the morbidity and mortality profile of these individuals.

Nurses need the skill and expertise to recognize clinical characteristics, response to treatment and its management so that they can provide appropriate care in this population ^(^ ^33^ ^-^ ^34^ ^)^ . While measures of quality of care towards HF are reported only in patients hospitalized for HF, some measures seem to be beneficial for all patients, regardless of the cause of hospitalization ^(^ ^35^ ^)^ . HF is a chronic condition, with high cost and complex treatment because it has multiple factors involved ^(^ ^11^ ^)^ . It should, therefore, be adequately managed with a view to better control, reduction of morbidity and improvement in quality of life.

This study has as a strength the disclosure of which are the clinical characteristics of HF associated with the greater chance of FD and that, therefore, can be considered as focus of attention of the nursing care. To our knowledge, this seem to be the first study to analyze each component of HF by associating it with the occurrence of FD. The identification of risk factors for early decompensation, recognition and treatment can prolong life with better quality, reduce costs and reduce risks for FD ^(^ ^2^ ^,^ ^30^ ^,^ ^36^ ^-^ ^38^ ^)^ . Caring for an elderly with HF implies understanding their perception and experience with the disease, evaluating its repercussions and the elderly’s autonomy to perform BADL, stimulating their potentialities and offering support so that they perceive, in everyday experience, ways of self-care.

This investigation has limitations. Firstly, it was not possible to estimate etiology insofar as the study was carried out in cross-section. For the number of variables examined in the present study, a larger sample would have allowed for more in-depth analyzes, such as the adjustment of the analyzes to confounding variables. In addition, the non-follow-up of the participant in the period prior to admission limited the FD analysis, since it was estimated retrospectively. Finally, longitudinal studies are mandatory and will help determine the real impact of factors that predict functional decline in this population.

Despite the limitations, the results found serve to guide nursing practice in the care of the elderly with HF. Previous analyzes of elderly patients with HF lack evidence about the clinical characteristics, such as those described in this study, which were analyzed as a multifactorial syndrome. These results made it possible to characterize the functionality and its association with HF, which, to date, have not been well described in the literature, especially in Brazilian studies. Likewise, the present study has a critical value in planning care for the growing population of older people with HF in the Brazilian scenario.

From the identification of patients with difficulty with one or more BADL or having a progression in the dysfunction, it is possible to conduct a more complete evaluation and an individualized assistance ^(^ ^39^ ^)^ , not only by the nurse but by the entire health team. Thus, the results of this study serve as an indication that the elderly with HF should be carefully evaluated, since the repercussions of the clinical characteristics have a strong impact on the patient’s functionality since hospital admission and that can last after discharge.

## Conclusion

The prevalence of FD at hospital admission in the elderly with HF was 70.2%. In the admission period, 3.7% of the elderly with HF were totally dependent, 66.6% were partially dependent and 29.7% were independent for the BADL. The most frequent clinical characteristics of HF at hospital admission were dyspnea, fatigue, PND and palpitation; however, only dyspnea, edema of LL, cough, precordial pain and pulmonary crackling were associated with increased chance of FD at admission among elderly with HF.
